# Perrault Plus Syndrome Due to the LARS2 Variant c.457A>C Manifesting With Epilepsy, Cognitive Impairment, Myopathy, Spastic Tetraparesis, and Deafness: A Case Report

**DOI:** 10.7759/cureus.106193

**Published:** 2026-03-31

**Authors:** Josef Finsterer

**Affiliations:** 1 Neurology, Neurology and Neurophysiology Center, Vienna, AUT

**Keywords:** epilepsy, lars2, leucyl-trna synthetase-2, perrault syndrome, spasticity

## Abstract

To our knowledge, this is the first carrier of the homozygous variant c.457A>C in LARS2 who has not only Perrault syndrome but also additional neurological symptoms.

The patient is a 35-year-old man with a history of sensorineural hearing loss since birth, rare generalized tonic-clonic seizures, progressive gait disturbance and cognitive decline since the age of 33, and spasticity since the age of 34. Examination of his symptoms revealed a multisystem mitochondrial disorder due to the c.457A>C variant in LARS2, which manifested phenotypically as leukodystrophy, cognitive impairment, epilepsy, hearing loss, myopathy, and spastic tetraparesis. The patient benefited from drug treatment with levetiracetam (1000 mg/day) and a vitamin cocktail.

In summary, this case suggests an association between the homozygous variant c.457A>C in LARS2 and a severe, adult-onset, neurologic phenotype including leukoencephalopathy, cognitive decline, progressive spastic tetraparesis, myopathy, and epilepsy within the expanding LARS2 spectrum. Physicians should be aware that LARS2 variants can manifest as a multisystem disorder and not just as Perrault syndrome.

## Introduction

The LARS2 gene is located on chromosome 3p21.31 and encodes class 1 mitochondrial leucyl-tRNA synthetase, an enzyme that catalyzes the aminoacylation of specific tRNA with leucine, which is essential for protein synthesis in mitochondria [1]. Pathogenic variants in LARS2 disrupt mitochondrial protein synthesis and manifest phenotypically as Perrault syndrome, hydrops-lactic acidosis-sideroblastic anemia (HLASA) syndrome, and leukodystrophy [2]. Core clinical features of Perrault syndrome include sensorineural hearing loss (SNHL) in both sexes, ovarian insufficiency in females, either type 1 or type 2 diabetes, and neurologic involvement [2]. More rare features of LARS2 variants include facial dysmorphism, hypospadias, Marfanoid habitus and cleft palate, developmental delay, cognitive impairment, cerebellar ataxia, hyperkinesia, pyramidal signs, seizures, extrapyramidal symptoms, aggression, self-mutilation, uterine hypoplasia, mitochondrial myopathy, sensorimotor neuropathy, and muscle hypotonia [2-15]. HLASA syndrome is characterized by ascites, anemia, and lactic acidosis.

Beyond its central role in mitochondrial translation, abnormal leucyl-tRNA synthetase is also implicated in other conditions. Elevated LARS2 levels have been observed in the prefrontal cortex of patients with bipolar disorder, establishing a link between mitochondrial dysregulation and neuropsychiatric disorders [16]. Furthermore, epigenetic modifications at the LARS2 locus are associated with traits such as obesity, and certain coding variants have been linked to an increased risk of type 2 diabetes [17]. LARS2 also plays an important role in modulating cell function beyond energy production. In granulosa cells, reduced LARS2 expression has been shown to impair mitochondrial integrity, leading to decreased cell proliferation and increased apoptosis, which may underlie premature ovarian failure [18]. Furthermore, a specific subset of immune-regulatory B cells expressing LARS2 has been identified in colorectal cancer, suggesting that LARS2-mediated mitochondrial aminoacyl-tRNA biosynthesis may influence the phenotype of immune cells and the tumor microenvironment [19]. Leucyl-tRNA synthetase also has therapeutic relevance [20].

More than 20 pathogenic LARS2 variants have been described in 25 patients [2-15]. Perrault syndrome due to the c.457A>C variant in LARS2 has only been described in two patients so far [2,11]. We report an adult man with homozygous c.457A>C in LARS2 and severe leukoencephalopathy, epilepsy, cognitive decline, myopathy, and spastic tetraparesis, further delineating the LARS2‑associated neurologic spectrum.

## Case presentation

**Figure 1:**
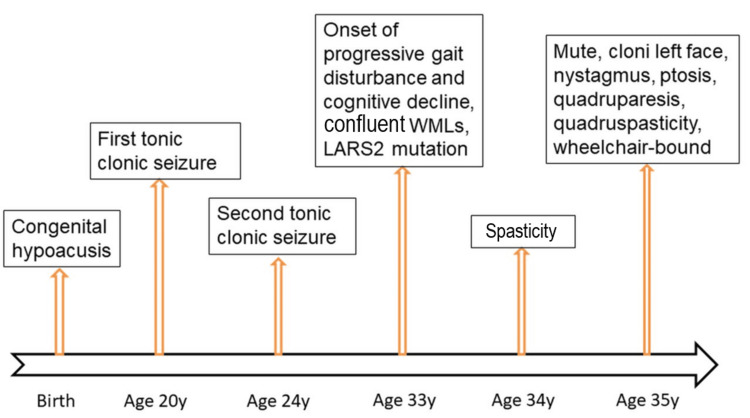
Disease course of the index patient. The x-axis shows the patient's age, while the y-axis shows the onset of the different manifestations WMLs: white matter lesions

The patient is a 35-year-old man, 167 cm tall, weighing 50 kg, who was diagnosed with Perrault syndrome at the age of 33 based on his clinical presentation and the pathogenic, homozygous variant c.457A>C (p.Asn153His) in LARS2 detected by whole exome sequencing. His medical history included normal psychomotor development (he had successfully completed primary and secondary school), SNHL since birth, and two generalized tonic-clonic seizures, the first at age 20 (treated with carbamazepine) and the second at age 24, progressive gait disturbance since the age of 33 (requiring a walking aid), cognitive deterioration since the age of 33, manifesting with noticeable changes in memory, language, attention, and executive function, and spasticity since the age of 34, requiring a wheelchair (Figure 1).

**Figure 2:**
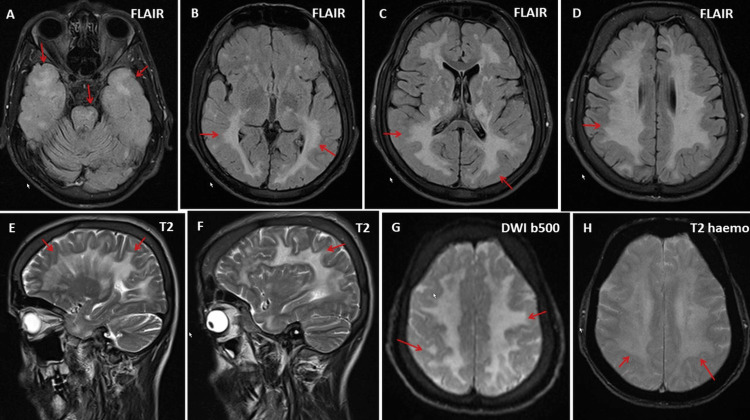
The MRI of the index patient shows extensive, supratentorial, patchy, confluent T2- and FLAIR-hyperintense areas (A-D). These are also hyperintense on DWI and T2 hemo-sequence (G-H). The basal ganglia are only minimally affected; the subcortical white matter is spared; a patchy pattern of involvement is also seen in the frontobasal and temporobasal regions. Additionally, small patchy, T2-hyperintense areas are found in the cerebellar peduncle and pons (E-F). The pons is atrophic, and there is evidence of incipient cerebellar atrophy MRI: magnetic resonance imaging; FLAIR: fluid-attenuated inversion recovery; DWI: diffusion-weighted imaging

Assessment of gait disturbance at the age of 33 using cerebral magnetic resonance imaging (MRI) revealed extensive, inhomogeneous, confluent signal abnormalities of the deep cerebral white matter and infratentorially, which initially led to a suspicion of leukodystrophy (Figure 2).

**Figure 3:**
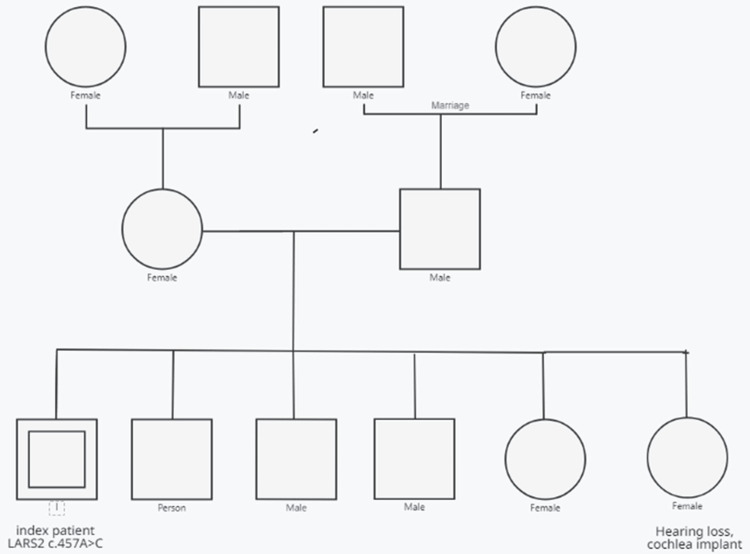
Pedigree of the index patient's family. The index patient was homozygous for c.457A>G. Only the index patient was genetically tested. Only one of the index patient's sisters presented with SNHL. All other family members were clinically unaffected SNHL: sensorineural hearing loss

There was also mild pontine and cerebellar atrophy. Nerve conduction studies revealed a slight proximal axonal lesion of the left peroneal nerve, but were otherwise unremarkable. Blood tests showed hypertriglyceridemia and iron deficiency. Creatine kinase (CK), lactate, pyruvate, and gonadotropins were within the normal range. The family history revealed SNHL in a sister, who was successfully treated with a cochlear implant (Figure 3). His maternal and paternal grandfathers were first cousins. No other family members have developed seizures, hearing impairment, cognitive impairment, muscle weakness, or spasticity.

A follow-up appointment was made at the age of 35 due to further deterioration of gait, cognition, and spasticity. The clinical neurological examination revealed a mute patient who was brought in a wheelchair and required full assistance to transfer to bed. He was alert and bradyphrenic, had hypoacusis, and performed simple tasks only irregularly. There was spontaneous nystagmus, and eye movement for horizontal gaze was irregularly impaired. Repeated clonus was observed in the left corner of the mouth and on the cheek. There were bilateral ptosis with right-sided emphasis, muscle weakness in the upper and lower extremities with right-sided emphasis, spastic tetraparesis with right-sided emphasis, and diffuse bilateral muscle atrophy. There were non-fixed flexion contractures in the right arm. The finger-snapping reflex was positive on both sides. The patellar tendon reflex was subclonic on the right side and normal on the left side. There was no response to pinching of the skin in any of the four limbs. The patient was unable to stand or walk without support.

**Figure 4:**
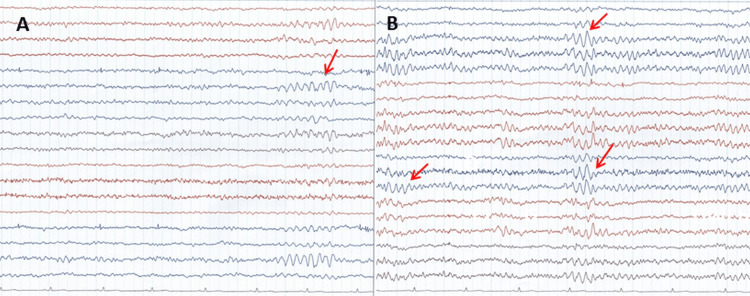
EEG recordings at age 35 show repeated generalized patterns of polymorphic, slow, and sharp waves with a duration of up to 1.5 seconds (A-B) EEG: electroencephalography

Blood tests only revealed mild vitamin D deficiency and iron deficiency. CK was normal, HbA1c was 4.4, and triglycerides were mildly elevated. Electrocardiography (ECG) and echocardiography were normal, and there was no renal or endocrine involvement. The follow-up MRI was unchanged from the previous imaging study. The Mini-Mental State Examination (MMSE) was 0. Needle electromyography was myogenic, without abnormal spontaneous activity. A full interference pattern could not be recorded. Electroencephalography (EEG) during the awake state showed background alpha activity with a frequency of 9-10 Hz extending to the frontal lobe. Repeated, generalized patterns of polymorphic, slow, and sharp waves (6/sec) with a duration of up to 1.5 seconds were recorded (Figure 4). Based on the clinical picture with facial clonus and the EEG, levetiracetam (1000 mg/day) was started. His current medication included levetiracetam, olanzapine (5 mg/day), iron, and vitamins.

## Discussion

The patient presented here is remarkable in several respects. To our knowledge, he is only the second patient to carry the c.457A>C variant in LARS2 in the homozygous form [2,11]. The first reported patient carrying the homozygous c.457A>C variant was a 27-year-old woman from Saudi Arabia with Perrault syndrome, born to consanguineous parents, described by Al-Jaroudi et al. in 2019, who phenotypically presented with a Marfanoid habitus, Tarlov cysts, congenital SNHL, hypergonadotropic hypogonadism, hypoplastic uterus, streak ovaries, primary amenorrhea, and degenerative changes in the spine [11]. The patient did not present with any neurological abnormalities. An MRI of the brain was not performed. The second patient carrying the c.457A>C variant in LARS2 was an eight-year-old female child from France who also carried the c.1565C>A variant in addition to the c.457A>G variant and was described by Carminho-Rodrigues et al. in 2020 [2]. Since the age of four, the patient had presented with SNHL, a missing left ovary, amenorrhea, astigmatism, and three café-au-lait spots [2]. The patient did not develop neurological abnormalities. The MRI of the brain was normal [2]. 

Second, the index patient behaved differently from the patient described by Al-Jaroudi et al. [11]. In addition to SNHL, the patient also suffered from epilepsy, cognitive decline, myopathy (ptosis, tetraparesis, myogenic electromyography), spasticity, and leukoencephalopathy. However, most of these neurological features reported in the index patient are not entirely novel for LARS2 in general, but their combination with homozygous c.457A>C in an adult man may extend the phenotype at the "severe neurologic" end. The different phenotype compared to that described by Al-Jaroudi et al. [11] is probably due to different residual enzyme activity. In addition, genetic, environmental, and epigenetic factors may have contributed to this phenotypic heterogeneity.

Third, the c.457A>C variant is not described in the gnomAD database, but has been classified once as "probably pathogenic" and once as "of unclear clinical significance" in ClinVar [2]. The amino acid change is predicted to be pathogenic by all algorithms used (pathogenicity prediction scores such as PolyPhen (Polymorphism Phenotyping), SIFT (Sorting Intolerant From Tolerant), MutationTaster, and CADD (Combined Annotation Dependent Depletion)). Since the c.457A>C variant alters the second nucleotide of exon 6, it may affect splicing, as predicted by dbscSNV (score: 0.93) and previously reported in homozygosity by Al-Jaroudi et al. [11]. Ultimately, the c.457A>C variant was classified as pathogenic according to the American College of Medical Genetics and Genomics (ACMG) criteria in the original report. The variant affects a residue that is completely conserved across all sequenced vertebrates and is most commonly reported in patients of Arabic ancestry.

The fourth point is that some of the additional phenotypic features of the index patient have been described previously in carriers of mutations in the LARS2 gene, but not in the two patients carrying the c.457A>C variant [2,11]. Epilepsy has been described in a single patient carrying the compound heterozygous LARS2 mutations c.1987C>T and c.371A>T [10]. Unfortunately, the type of seizures, treatment, and response to antiepileptic drugs were not described in detail [10]. Cognitive decline, as seen in the index patient, has not been reported, but psychomotor retardation has been described in one patient [13]. Myopathy has also been rarely described in carriers of LARS2 variants. Myopathy has been described in particular in a newborn girl with neck muscle weakness, in whom a muscle biopsy revealed complex I deficiency [3]. Spasticity was the most common feature in carriers of the LARS2 mutation, along with SNHL and hypogonadism. Spasticity was a prominent feature not only in the index patient but also in the patient described by Bayanova et al. [13] and the patient described by van der Knaap et al. [10].

Limitations of the study are that only a single case was reported, that no cerebrospinal fluid (CSF) examinations and no functional assays were performed, that mitochondrial work-up was incomplete, that other family members were not genetically tested, and that no extensive quantification of cognitive data was obtained. 

## Conclusions

This case suggests an association between the homozygous c.457A>C variant in the LARS2 gene and a severe, adult-onset neurological phenotype encompassing leukoencephalopathy, cognitive decline, progressive spastic tetraparesis, myopathy, and epilepsy within the expanding LARS2 spectrum. The phenotypic variability compared to previously described cases may reflect differences in genetic background, modifier genes, or incomplete penetrance. Clinicians should be aware that LARS2 variants can manifest not only as Perrault syndrome but also as a late-onset, multisystem disorder.
